# Locating Organizations and Their Methods in Registrations of Clinical Migraine Trials: Analysis of ClinicalTrials.gov

**DOI:** 10.3389/fneur.2021.739109

**Published:** 2021-10-01

**Authors:** Pengfei Zhang, Thien Phu Do

**Affiliations:** ^1^Department of Neurology, Robert Wood Johnson University Hospital - New Brunswick, New Brunswick, NJ, United States; ^2^Department of Neurology, Danish Headache Center, Faculty of Health and Medical Sciences, Rigshospitalet Glostrup, University of Copenhagen, Copenhagen, Denmark

**Keywords:** migraine research, clinical trial design, clinical research, migraine, headache

## Abstract

**Background and Objective:** ClinicalTrials.gov is a centralized venue for monitoring clinical research and allows access to information on publicly and privately funded studies. To better recognize influential institutions in the field of headache, we identified major organizations conducting clinical trials in migraine research. Furthermore, we examined the frequency of different study designs.

**Methods:** Utilizing the ClinicalTrials.gov application programming interface, we extracted studies including individuals with migraine from February 29, 2000, to July 28, 2020, for the following: (1) host organization, (2) study type, (3) primary purpose, (4) intervention model, and (5) allocation.

**Results:** We included 921 entries encompassing 423 organizations. Thirty-two organizations produced ≥5 entries each and 40.0% of all entries. Most, 86%, were interventional studies while 13.6% were observational studies. The most common study design had a randomized allocation of participants. The most frequent primary purpose was treatment (62.4%) followed by prevention (13.0%). There were 56.9% parallel assignment models, 15.2% single group assignment models, and 12.4% crossover assignment models.

**Conclusion:** A minority of organizations contribute to a significant number of registrations of clinical migraine trials, suggesting that clinical research in migraine is oligarchic. The most common study is interventional and randomized, with parallel assignment of participants with treatment as the primary purpose. This likely reflects the need to evaluate novel putative pharmacological medications.

## Background

Migraine directly affects more than 1 billion people worldwide and incurs significant individual and societal burden (1). Current standard of care includes both acute and preventive medications (2). The past decade has experienced great therapeutic advances in treatment of migraine including introduction of monoclonal antibodies targeting calcitonin gene-related peptide (CGRP) or its receptor, small-molecule CGRP receptor antagonists, and ditans (3). Even so, there are many unmet needs as not all individuals are treatment responders to existing medications, and clinical trials evaluating other putative compounds are necessary (4). Assessing efficacy and tolerability is essential during drug development, and prospective, randomized, controlled clinical trials remain the gold standard.

ClinicalTrials.gov is a centralized database for monitoring clinical research. Created from the US Food and Drug Administration Amendments Act of 2007, ClinicalTrials.gov provides patients and healthcare professionals access to federal clinical trial registry of investigational drug applications for publicly and privately funded studies (5). This allows for large-scale analysis of characteristics of clinical trials (6). The aim of this study was to identify influential organizations conducting clinical trial research in migraine as well as to identify the frequency of different study designs in clinical trials in migraine.

## Methods

We downloaded profiles of trials including subjects with migraine on ClinicalTrials.gov from its inception, February 29, 2000, to July 28, 2020, to analyze the following: (1) host organization; (2) study type; (3) primary purpose; (4) intervention model; and (5) allocation. Our study consisted of four phases.

### Initial Exploratory Phase

Prior to any data extraction, we had to determine how many registration entries exist in the database. We conducted an exploratory inquiry to identify the number of entries containing the word “migraine” through the following search:

https://clinicaltrials.gov/api/query/full_studies?expr=migraine&fmt=JSON&min_rnk=4000.

This was equivalent to entering “migraine” into the “other terms” search field in the ClinicalTrials.gov main page and obtaining the first 4,000 results. If this search returned ≤4,000 entries, then we would have identified all the relevant entries.

### Data Access Phase

After identifying all relevant entries, we extracted these entries through the ClinicalTrials.gov API through Clojure, a functional programming language.

### Inclusion and Exclusion Phase

**Figure 1:**
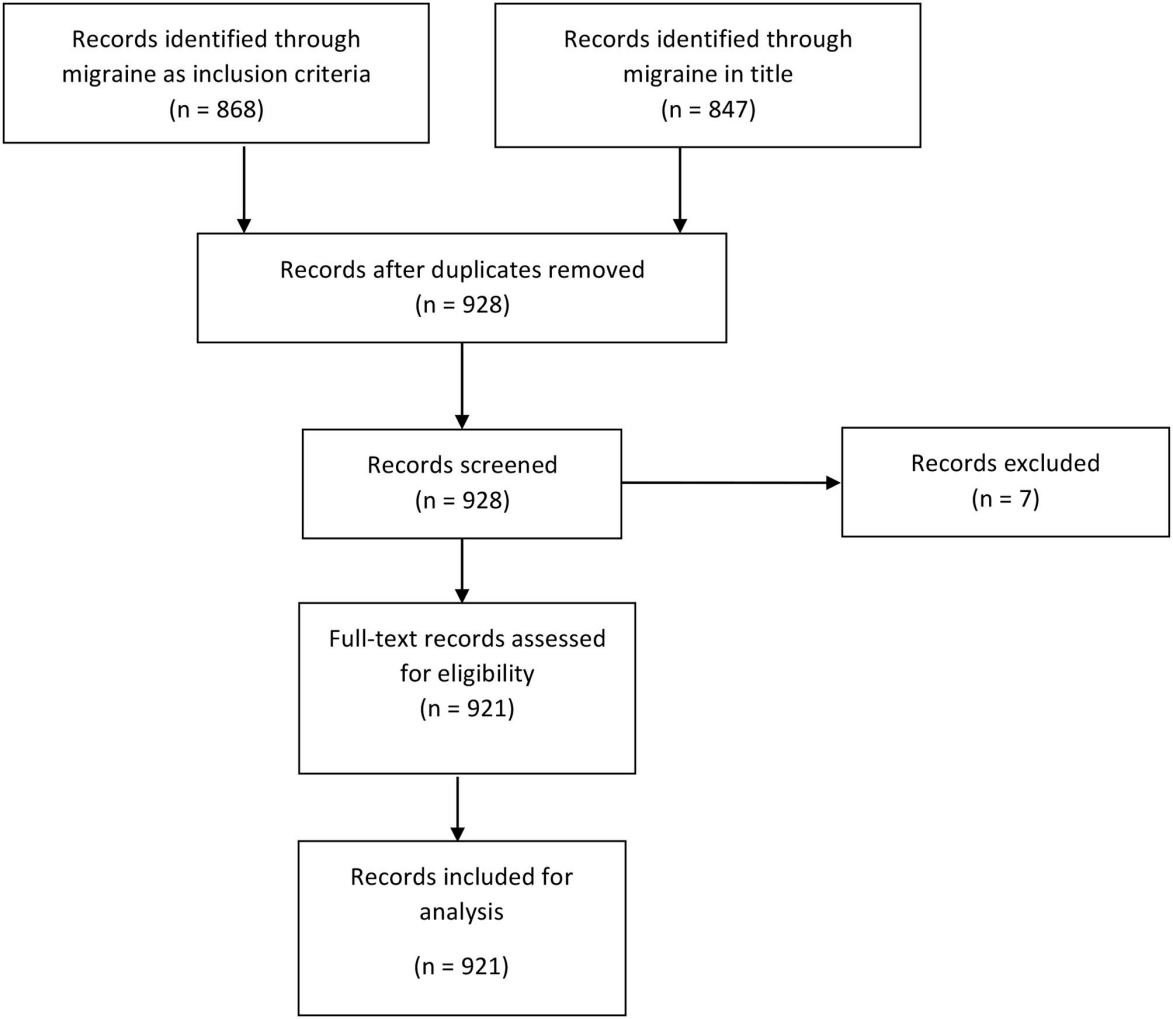
Flow diagram of selection of registrations on ClinicalTrials.gov.

We used the PRISMA paradigm for inclusion and exclusion of trials in our search strategy (Figure 1). For the identification phase, we screened trials through two arms: (1) If a trial contained the word “migraine” or “migraines” in the inclusion criteria under the “Eligibility Criteria” field, then we included it. (2) If a trial contained the word “migraine” or “migraines” in the title—whether the “Brief Title” field and/or the “Official Title” field—then it was included.

Once the two arms were identified, they were merged and duplicates were removed. The records were then screened and removed, with reasons, as well as assessed for eligibility. The inclusion criterion was at least one arm including subjects with migraine.

### Data Analysis Phase

We used custom backend codes in Haskell to identify the following:

1. “Responsible Organization”—i.e., “Lead Sponsor” designated by “LeadSponsorName” field in each entry.
2. “Study Type”—i.e., “interventional,” “observational,” “expanded access.”
3. “Design Primary Purpose”-designated by either “treatment,” “prevention,” “basic science,” “supportive care,” “diagnostic,” “health services research,” “educational/counseling/training,” “screening,” device feasibility,” or “other.” Of note, “prevention” study designation is not prophylactic treatments of migraine but defined as “preventing the development of a specific disease or health condition” (7).
4. “Design Intervention Model.”-The options were “parallel assignment,” “single group assignment,” “factorial assignment,” “sequential assignment,” or “crossover assignment.”
5. “Design Allocation Count.”-The options were “randomized,” “non-randomized,” or “n/a.”
6. “Overall Status.”-The options were “completed,” “recruiting,” “unknown status,” “terminated,” “not yet recruiting,” “withdrawn,” “active, not recruiting,” “enrolling by invitation,” “suspended,” and “no longer available.”
7. “Clinical Trial Phase.”-The options ranged from “early phase 1” studies to “phase 4” studies.

Of note, not all study entries identify the “Study Type,” “Design Primary Purpose,” “Design Intervention Model,” and “Design Allocation Count.” All entries should include the full name of the organization.

## Results

The exploratory search inquiry identified 868 entries containing “migraine(s)” in the inclusion criteria and 857 containing “migraine(s)” in the title. There were 787 duplicates that contained both “migraine” in the title and “migraine.” After removal of duplicates, there were 928 unique entries. During the screening process, we excluded 7 entries. Our final data set consisted of 921 unique entries (Figure 1).

### Responsible Organization

We identified 423 unique trial organizations encompassing 921 studies. Fifty organizations registered for four or more studies, 28 registered for three studies, and 52 registered for two studies. The remaining 293 organizations (293/423 or 69.2%) registered only one study, accounting for 31.8% (293/921) of all studies included.

Thirty-two organizations produced ≥5 entries encompassing 40.0% (368/921) of all entries (Table 1). There were 15 academic organizations in the top 32, producing a total of 138 entries. There were 17 non-academic organizations on the top 32, producing 230 entries. Overall, the organizations accounting for most entries were The Danish Headache Center, Merck, and Allergan. Other major academic centers such as Thomas Jefferson University, Montefiore Medical Center, and Johns Hopkins University follow.

**Table 1:** Ranking of organizations with ≥5 registrations related to migraine on ClinicalTrials.gov.

**#**	**Organization**	**Number of entries** **(*n* = 921)**
1	Danish Headache Center	42 (4.6%)
2	Merck Sharp & Dohme Corp.	31 (3.4%)
3	Allergan	30 (3.3%)
4	Eli Lilly And Company	27 (2.9%)
5	GlaxoSmithKline	23 (2.5%)
6	Amgen	21 (2.3%)
7	Thomas Jefferson University	17 (1.8%)
8	Montefiore Medical Center	12 (1.3%)
9	Johnson & Johnson Pharmaceutical Research & Development LLC	11 (1.2%)
10	Biohaven Pharmaceuticals Inc.	10 (1.1%)
11	Pfizer	9 (0.9%)
12	Johns Hopkins University	9 (0.9%)
13	Roger Cady MD	9 (0.9%)
14	Teva Branded Pharmaceutical Products R&D Inc.	8 (0.9%)
15	Novartis Pharmaceuticals	8 (0.9%)
16	Mayo Clinic	8 (0.9%)
17	Dr. Reddy's Laboratories Limited	8 (0.9%)
18	University Of California San Francisco	7 (0.8%)
19	NYU Langone Health	7 (0.8%)
20	Electrocore Inc	7 (0.8%)
21	Theranica	6 (0.7%)
22	Glostrup University Hospital Copenhagen	6 (0.7%)
23	Cefaly Technology	6 (0.7%)
24	Alder Biopharmaceuticals Inc.	6 (0.7%)
25	Wake Forest University Health Sciences	5 (0.5%)
26	University Of Liege	5 (0.5%)
27	Universidade Federal De Pernambuco	5 (0.5%)
28	The Cleveland Clinic	5 (0.5%)
29	Nupathe Inc.	5 (0.5%)
30	Norwegian University Of Science And Technology	5 (0.5%)
31	Massachusetts General Hospital	5 (0.5%)
32	Abbott Medical Devices	5 (0.5%)

*A total of 921 registrations were identified. The top 32 organizations account for 368/921 (40%) of registrations*.

### Study Type

For study type, 86.2% (794/921) were interventional studies, 13.6% (125/921) were observational studies, and 0.2% (2/921) expanded access studies. There were no missing entries.

### Design Allocation

For design allocation, 69.1% (636/921) were randomized studies, 6.9% (64/921) were non-randomized studies, and 9.9% (91/921) were N/A. This sums to 791; thus, there were 14.1% (130/921) studies that did not contain this label and were unaccounted for.

### Design Primary Purpose

For design primary purpose, there were 62.4% (576/921) treatment studies, 13.0% (120/921) prevention studies, 3.8% (35/921) basic science studies, 0.1% (9/921) supportive care studies, 0.9% (8/921) diagnostic studies, 0.7% (6/921) health services research studies, 0.3% (3/921) educational/counseling/training studies, 0.4% (4/921) screening studies, and 0.1% (1/921) device feasibility study. Twenty studies were labeled as “other.” This amounted to 782; thus, there were 15.1% (139/921) studies that did not contain this label and were unaccounted for.

### Design Interventional Model

For the design interventional model, there were 56.9% (524/921) parallel assignment models, 15.2% (140/921) single group assignment models, 12.4% (114/921) crossover assignment models, 1.2% (11/921) factorial assignment models, and 0.5% (5/921) sequential assignment models. This aggregated to 794 studies; there were 127 studies that did not contain this label and were unaccounted for.

Of note, we reviewed the 42 studies registered by the Danish Headache Center, the highest ranking organization in our list. Of the registrations, 21 were crossover assignment and 8 studies were in parallel assignments. (The others were of different design or were unclassified).

Of note, some of the studies included detailed descriptions of the design interventional model in addition to labeled as one of the above categories. These descriptions themselves were not considered here.

### Overall Status

There were 61.5% (566/921) “completed” trials, 11.3% (104/921) “recruiting” trials, 8.7% (80/921) “unknown status” trials, 5.5% (51/921) “terminated” studies, 5.3% (49/921) “not yet recruiting” studies, 3.3% (30/921) “withdrawn” studies, 2.9% (27/921) “active, not recruiting” studies, 0.9% (8/921) “enrolling by invitation” studies, 0.4% (4/921) “suspended” studies, and 0.2% (2/921) “no longer available” studies.

### Clinical Trial Phase

There were 0.7% (6/921) “early phase 1” trials, 3.9% (36/921) “phase 1” trials, 10.2% (94/921) “phase 2” trials, 13.5% (124/921) “phase 3” trials, and 11.4% (105/921) “phase 4” trials. Therefore, 60.4% (556/921) studies did not include a phase designation.

## Discussion

We demonstrate that the landscape for clinical research in migraine is oligarchic; 3% of organizations were responsible for about 40% of registrations on ClinicalTrials.gov. Furthermore, academic organizations accounted for just under half of the 32 most productive organizations. Our list of influential headache research institution is similar to a recent scientometric study on the most productive academic institution in headache (8). Taken together, these data suggest that a limited number of well-known academic centers as well as a small number of pharmaceutical organizations are responsible for the majority of our knowledge on migraine pathophysiology and treatment. Indeed, a majority of researchers in the world hold limited influence over the shaping of our field through clinical research. About 70% of organizations had only one study registered, encompassing only 30% of total registrations.

Clinical trial registrations in migraine research comprise mostly of interventional studies with a parallel assignment model. Since vigorous efficacy, tolerability, and safety data are a requirement from regulatory organs such as U.S. Food and Drug Administration and European Medicines Agency for approval of novel therapies, it is not surprising that most registrations of clinical trials were intervention studies. Although a randomized and parallel assignment design is the gold standard in drug development, these types of studies are expensive and cumbersome to conduct (9–11). Since public funding is limited, and therefore competitive, while private funding is available primarily to pharmaceutical organizations, the clinical trial landscape inevitably favors a limited number of institutions or industrial stakeholders.

As in other fields, few academic institutions in our field appeared to achieve their position through reputation. For example, the Danish Headache Center was the highest ranked organization. Likely, this is due to the center being one of the first to offer a multidisciplinary approach collected under one institution (12). Indeed, organizations with a longer tradition of migraine research accounted for the majority of registrations. Nonetheless, not all organizations with a history of clinical migraine research were in the top 32 in our list. One explanation is that researchers at one organization often serve as principal investigators in studies sponsored by another organization; in these cases, the latter becomes the “lead” sponsor. Other explanations include registrations under different names or lack of registrations of conducted clinical trials. If the reason is the latter, organizations should aim to improve pre-registrations to increase transparency and to reduce bias in clinical studies.

It is surprising that most clinical trials did not contain a clinical trial phase designation. Presumably, these accounts for trials that were experimental or observational in nature. A future study may be able to elucidate this further. Moreover, approximately 9% of trials appeared to be either “terminated,” “withdrawn,” “suspended,” or “no longer available;” future studies to identify why these trials were terminated would be valuable for clinical trial research design. As a future direction, it would be interesting to ClinicalTrials.gov for determining prevention vs. treatment studies are accurately followed. Finally, a future study should assess whether data from all registered clinical trials are published and publicly available to increase transparency.

### Strength and Limitations

ClinicalTrials.gov is an influential database. Each study is uniquely designated, allowing for the study of clinical trial characteristics otherwise not possible through traditional literature reviews. However, we did not include other regional databases such as the European Union Clinical Trial Registry or Japan Primary Registries Network; therefore, it is unclear how our results were biased toward, studies conducted in the US. However, it is worth noting that a significant number of our entries were not from American organizations.

CinicalTrials.gov, although comprehensive, may suffer from poor reporting of clinical trial results as well as exclusions of basic science research (6). Furthermore, multiple organizations may participate in one clinical trial, but only one organization was listed as primary. In addition, based on our inclusion criteria, studies involving only healthy volunteers were not included. It is also unclear how retrospective registration affects our data.

## Conclusion

Clinical research in migraine is dominated by the top 3% of organizations accounting for almost half of all migraine registrations on ClinicalTrials.gov. The most common registration was a clinical study that is interventional and randomized, with parallel assignment for treatment purpose study design.

## Data Availability Statement

The raw data supporting the conclusions of this article will be made available by the authors, without undue reservation.

## Author Contributions

PZ and TD conceptualized and designed the study. PZ conducted data collection. All the authors interpreted the data, contributed to the drafting of the manuscript, and approved it to be published. All authors agreed to be accountable for all aspects of the work for any issue related to the accuracy or integrity of any part of the work. PZ attests that all listed authors meet authorship criteria and that no others meeting the criteria have been omitted.

## Funding

Rutgers University supplied funding for publication fees.

## Conflict of Interest

PZ has received honorarium from Lundbeck Biopharmaceuticals, Board Vitals, and Fieve Clinical Research. He collaborates with Headache Science Incorporated without receiving financial support. He has ownership interest in Cymbeline LLC. The remaining author declares that the research was conducted in the absence of any commercial or financial relationships that could be construed as a potential conflict of interest.

## Publisher's Note

All claims expressed in this article are solely those of the authors and do not necessarily represent those of their affiliated organizations, or those of the publisher, the editors and the reviewers. Any product that may be evaluated in this article, or claim that may be made by its manufacturer, is not guaranteed or endorsed by the publisher.

## Abbreviations

API: application programming interface
PRISMA: Preferred Reporting Items for Systematic Review and Meta Analysis.
